# Corrigendum: A compact multi-functional model of the rabbit atrioventricular node with dual pathways

**DOI:** 10.3389/fphys.2023.1219583

**Published:** 2023-06-02

**Authors:** Maxim Ryzhii, Elena Ryzhii

**Affiliations:** ^1^ Department of Computer Science and Engineering, University of Aizu, Aizu-Wakamatsu, Japan; ^2^ Department of Anatomy and Histology, Fukushima Medical University, Fukushima, Japan

**Keywords:** atrioventricular node, rabbit heart model, aliev-panfilov model, dual pathway, conduction curve, coupling asymmetry, retrograde conduction, laddergram

In the published article, there was an error in Figure 9 as published. The position of tick bars on some axes in the **figure** was incorrect, there was a mistake in the *x*-axis titles in panels A, and the legends in panels B were wrong. The corrected Figure 9 and its caption “Comparison of experimental (Reid et al., 2003) (left panels) and simulated (right panels) anterograde and retrograde recovery curves in the control case and after FP and SP ablations. **(A)** Experimental recovery curves in the control case and after FP ablation and simulated recovery curves. **(B)** Experimental recovery curves in the control case and after SP ablation and simulated recovery curves. Dashed lines correspond to the simulated retrograde conduction in the control case without coupling asymmetry” appear below.

**FIGURE 9:**
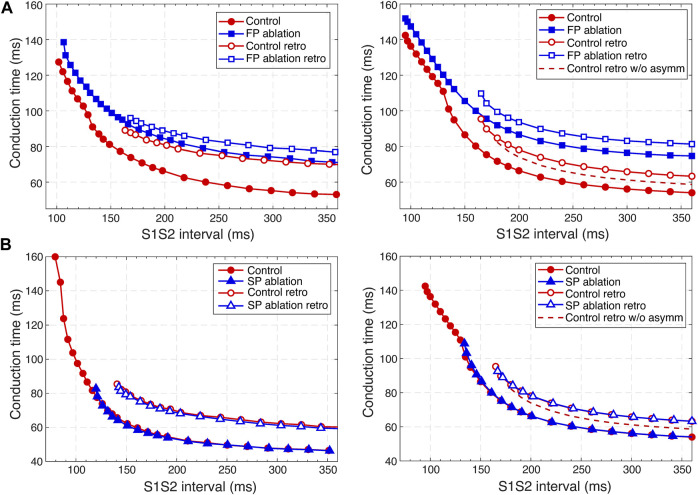
Comparison of experimental (Reid et al., 2003) (left panels) and simulated (right panels) anterograde and retrograde recovery curves in the control case and after FP and SP ablations. **(A)** Experimental recovery curves in the control case and after FP ablation and simulated recovery curves. **(B)** Experimental recovery curves in the control case and after SP ablation and simulated recovery curves. Dashed lines correspond to the simulated retrograde conduction in the control case without coupling asymmetry.

The authors apologize for this error and state that this does not change the scientific conclusions of the article in any way. The original article has been updated.

