# Supplementary material for: A compact multi-functional model of the rabbit atrioventricular node with dual pathways
Source: Front Physiol. 2023 Mar 10;14:1126648. doi: 10.3389/fphys.2023.1126648 (PMC10036810; doi:10.3389/fphys.2023.1126648)
Supplement: Supplementary file 1 [file DataSheet2.PDF]

Supplementary Material

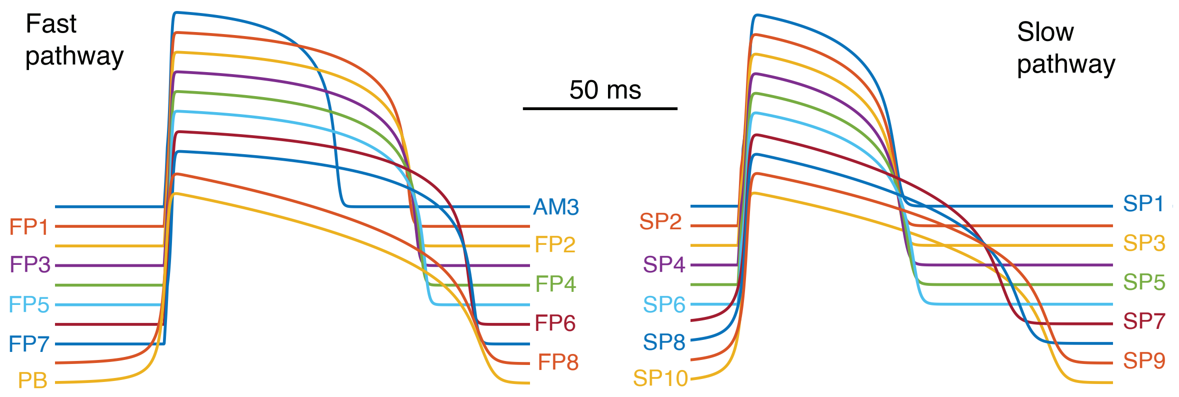

Figure S1. Action potentials profiles of the uncoupled model cells in the fast and slow pathways.
