# Supplementary material for: A compact multi-functional model of the rabbit atrioventricular node with dual pathways
Source: Front Physiol. 2023 Mar 10;14:1126648. doi: 10.3389/fphys.2023.1126648 (PMC10036810; doi:10.3389/fphys.2023.1126648)
Supplement: Supplementary file 2 [file DataSheet1.PDF]

Supplementary Material

Table S1. Parameters of the model, first and second rows.

	SN	PS1	PS2	PS3	AM1	AM2	AM3	FP1	FP2	FP3	FP4	FP5	FP6	FP7	FP8	PB	HB1	HB2	HB3	HB4	HB5	HB6
**First row**																						
a_1	-0.0694	0.08	0.13												-0.0368		0.13					
a_2	0.20	0.16	0.13												0.28		0.13					
μ_1	0.16	0.18	0.20		0.22			0.15					0.14		0.065	0.060	0.02					
μ_2	4.2		3.3	2.5	1.6			1.7	1.8	1.85	1.95	2.05	2.15	2.25	4.6		0.35	0.1				
k	9		10	14	18								15		8		10					
ε_0	0.04	0.036	0.025		0.018			0.011							0.042		0.042	0.050				
**Second row**																						
						AM*	SP1	SP2	SP3	SP4	SP5	SP6	SP7	SP8	SP9	SP10						
a_1	0					0.13							-0.0369		-0.0368		0					
a_2	0					0.13							0.28				0					
μ_1	0					0.22	0.23	0.22	0.215	0.205	0.190	0.175	0.07		0.06		0					
μ_2	0					1.6	1.8						2.5	3.3	3.7	3.9	0					
k	0					20	11						8				0					
ε_0	0					0.018	0.060						0.044		0.042		0					

Table S2. Coupling and asymmetry coefficients: first row, second row, and vertical.

	SN	PS1	PS2	PS3	AM1	AM2	AM3	FP1	FP2	FP3	FP4	FP5	FP6	FP7	FP8	PB	HB1	HB2	HB3	HB4	HB5
**First row**																					
d_x	41	45	47	56	68	95	95	84	70	64	62	60	59	59	55	80	50	50	48	48	48
α	1.0	1.0	1.0	1.0	0.75	0.53	0.62	0.75	0.80	0.85	0.95	1.0	1.25	1.40	1.50	0.65	0.70	1.0	1.0	1.0	1.0
**Second row**																					
						AM*	SP1	SP2	SP3	SP4	SP5	SP6	SP7	SP8	SP9	SP10					
d_x	0						59	57	57	56	56	53	32	32	32	0					
α	0						1.0									0					
**Vertical**																					
d_y	0					90	69	0								42	0				
α_y	0					1.0	1.0	0								1.0	0				

$c_t = 1000/4\ \mathrm{s}^{-1}$ (for MATLAB ODE solver)
